# Preparation and Safety Evaluation of *Centella asiatica* Total Glycosides Nitric Oxide Gel and Its Therapeutic Effect on Diabetic Cutaneous Ulcers

**DOI:** 10.1155/2022/1419146

**Published:** 2022-03-25

**Authors:** Yi-qiu Liu, Dandan Zhang, Junyu Deng, Ye Liu, Wei Li, Xuqiang Nie

**Affiliations:** ^1^College of Pharmacy, Zunyi Medical University, Zunyi 563006, China; ^2^Key Laboratory of Basic Pharmacology of Ministry of Education and Joint International Research Laboratory of Ethnomedicine of Ministry of Education, Zunyi 563006, China; ^3^Pharmacy Department, The Third Affiliated Hospital of Zunyi Medical University (The First People's Hospital of Zunyi), Zunyi 563099, China

## Abstract

Diabetic cutaneous ulcers (DCU) are a chronic and refractory complication of diabetes mellitus, which can lead to amputation or even death in extreme cases. Promoting the early healing of DCU and reducing the disability rate and treatment cost are important research topics in treating with integrated traditional Chinese and Western medicine. *Centella asiatica* total glycosides are extracted from the traditional Chinese medicine *Centella asiatica* and have angiogenic, anticancer, antioxidant, and wound healing effects. Nitric oxide (NO) is a critical component of wound healing. During the development of DCU, endogenous NO secretion is insufficient. It has been reported that exogenous nitric oxide can promote wound healing, but it is difficult to adhere to the skin because of its short half-life. Therefore, in this study, we used the polymer excipient hydroxyethyl cellulose as the matrix, combined with *Centella asiatica* total glycosides and NO, and developed a new type of topical gel that can promote wound healing. At the same time, we made a comprehensive research and evaluation on the preparation technology, quality standard, skin toxicity, reproductive toxicity, and pharmacodynamics against diabetic skin ulcers of the gel. According to our research results, the combination of C*entella asiatica* total glycosides and nitric oxide can accelerate the healing speed of DCU wounds, and 8% C*entella asiatica* total glycosides nitric oxide gel (CATGNOG) has the best effect in ulcer wound healing. CATGNOG has the advantages of feasible preparation method, controllable quality, good stability at low temperature, and no apparent skin toxicity and reproductive toxicity. It can effectively inhibit the growth of bacteria on the wound surface, relieve the inflammatory reaction of the wound surface, and promote the healing of ulcer wound, which provides a basis for further research of the preparation in the future.

## 1. Introduction

Diabetic cutaneous ulcers (DCU) are the main end-stage complication of diabetes mellitus, with considerable morbidity and mortality. In recent years, DCU has become a major clinical issue. The wound healing of DCU patients is impaired, resulting in chronic wounds, which eventually lead to amputation and even death. According to statistics, 15% of diabetic foot ulcers cause a lower limb amputation, and 85% of lower limb amputees have foot ulcers before [1] amputation [2]. The mortality rate associated with diabetic foot ulcers within 12 months is 5%, and the 5-year mortality rate is 42% [3]. Corresponding to high morbidity and mortality is the high cost of treatment, with 25%–50% of the total cost of diabetes treatment directly associated with DCU [4]. The nursing costs of patients with diabetic foot ulcers after one year of illness are 5.4 times higher than those of diabetic patients without foot ulcers, and the treatment costs of the highest grade ulcers are eight times higher than those of the lower grade ulcers [5], placing a heavy burden on patients, healthcare systems, and society. Skin wound healing is a complex dynamic process used to repair damaged cell structures and tissues. Traditionally, it can be divided into four overlapping stages: hemostasis, inflammation, proliferation, and maturation/remodeling [6]. These stages fail to happen in time and gradually, resulting in pathological wound healing disorders. The main reasons for refractory DCU include (1) lack of neovascularization in the wound; (2) stubborn bacterial infection in the wound; (3) skin dermal cells damaged or poorly differentiated. However, up to now, the therapeutic effects, including cell growth-stimulating factor, angiogenesis stimulating factor, and new antibiotics, are not significant. In this study, DCU was taken as the research object to study the healing of skin ulcers.

*Centella asiatic*a (L.) Urb., also known as Gotu Kola, is the whole dried plant of *Centella asiatica*, a member of the Umbelliferae family. *Centella asiatica* has a wide range of biological activities, which is widely used as a traditional Chinese medicine to treat various diseases, promote wound healing, and inhibit scar formation [7–9]. It has good efficacy in protecting the liver [10], improving the antitumor [11] antioxidant activities [12], treating cardiovascular diseases [13], protecting the nervous system, and treating anti-Alzheimer's disease [14]. Asiaticoside and madecassoside are white needle-like crystals of triterpenoid saponins extracted from *Centella asiatica*, which have rich pharmacological activity and favorable clinical effects and are the main active components of *Centella asiatica* total glycosides. Asiaticoside and madecassoside have been found to promote wound healing and to inhibit scar formation, with unique dual efficacy [15, 16]. It promotes wound healing by epithelialization. The mechanism includes increased migration of epithelial cells, the proliferation of fibroblasts, and new capillaries [17]. The proliferation of fibroblasts is closely related to skin repair. Studies have shown that asiaticoside can promote the proliferation of fibroblasts and migration and secretion of collagen [18, 19]. Neovascularization provides adequate nutrition for the proliferation of granulation, and the administration of *Centella asiatica* total glycosides can increase the expression of monocyte chemoattractant protein-1 (MCP-1) in keratinocytes as well as interleukin-1 *β* (IL-1 *β*) in macrophages, stimulate the production of vascular endothelial growth factor (VEGF), and promote angiogenesis [20]. In the animal burn model *in vivo*, *Centella asiatica* total glycosides positively affect the proliferation and cell growth of wound healing by stimulating collagen synthesis, reducing the oxidative stress of wounds, and inducing vasodilation [21]. Recently, phase III trial data and drug mechanism of ON101, a new drug for diabetic foot, have been published [22]. ON101 shows a much better clinical effect than the current standard treatment and nursing, which can significantly reduce the nursing cost of sugar foot wound ulcers and reduce the risk of amputation. The main ingredient, S1 *Centella asiatica* extract, promotes wound healing by increasing collagen synthesis, stimulating fibroblast proliferation and keratinocyte migration. Based on the detoxification, detumescence, and granulation effects of *Centella asiatica* and its clinical pharmacological effects on skin repair, we speculate that *Centella asiatica* total glycosides may contribute to the treatment of DCU disease.

Nitric oxide (NO) is a free radical gas produced by nitric oxide synthase (NOS), which has various biological activity signals and participates in various physiological and pathological reactions [23, 24]. A large number of studies have shown that NO has complex biological effects, and its role in dilating blood vessels and inhibiting platelet aggregation, antitumor, antibacterial, and neurotransmitter has been reported. NO plays a vital role in every stage, from inflammation to tissue remodeling [25, 26]. NO is involved in regulating immune responses and has a wide range of antibacterial activities [27]. NO is an important messenger molecule for wound healing, which regulates the activity of various growth factors and cytokines [28, 29]. During the proliferative phase, NO promoted the proliferation and differentiation of keratinocytes, endothelial cells, fibroblasts, and epithelial cells, improved local blood microcirculation, and promoted angiogenesis [30, 31]. Due to the important role of NO in wound healing, NO-based therapies for diabetic skin ulcers have received increasing attention in recent years. *In vivo*, NO is oxidized by nitric oxide synthase using arginine and molecular oxygen as substrates. There are three independent isoforms of NO synthase (NOS), namely, neural NOS (nNOS or NOS 1), inducible NOS (iNOS or NOS 2), and endothelial NOS (eNOS or NOS 3). The skin tissue mainly expresses eNOS, and the balance of NO in skin tissue is maintained by eNOS. iNOS can promote the healing of ulcer wounds by regulating inflammatory cells in wounds, while eNOS can promote the healing of ulcer wounds by promoting angiogenesis [32, 33]. It was found that the contents of eNOS and NO in skin tissue decreased obviously after wound formation, which led to vascular endothelial dysfunction, hindered wound healing, and increased NO to promote wound healing. The content of NO is directly related to wound healing [34, 35]. Therefore, the sustained role of exogenous NO in wound repair is a crucial scientific issue.

NO and asiaticoside have been studied in the treatment of wound healing, but the combined application of NO and asiaticoside in the study of skin ulcers at home and abroad has not been found [21, 36–38]. Exogenous NO is exposed in the air for a short time, which is easily oxidized by O_2_ in the air, producing toxic NO_2_ and losing its therapeutic ability. In this study, a new gel was prepared by combining *Centella asiatica* total glycosides with exogenous NO, which had the following conditions: (1) it maintains the slow release of NO on the wound; (2) it can be used as a covering surface for wound healing; (3) it has good biocompatibility and biodegradability; (4) the gel itself has therapeutic effects such as promoting wound healing and anti-infection.

## 2. Materials and Methods

### 2.1. Materials

Hydroxyethyl cellulose (3400–5000 mPa.s, 25°C), azone, glycerol, triethanolamine, and ethyl p-hydroxybenzoate were purchased from Aladdin-Reagent Co. Ltd. (Shanghai, China). *Centella asiatica* total glycosides (47.95%), asiaticoside standard (≥98% pure), and madecassoside standard were obtained from Pusi Biotechnology Co., Ltd. (Chengdu, China). *β*-Cyclodextrin, propylene glycol, sodium hydroxide, polyethylene glycol, and absolute ethanol were purchased from Sinopharm Chemical Reagent Co., Ltd. (Shanghai, China). Acetonitrile was purchased from Tedia (Tedia, Fairfield, OH, USA). 2,4-Dinitrochlorobenzene was purchased from Huamai Technology Co., Ltd. (Wuhan, China). Recombinant human epidermal growth factor solution was purchased from Shenzhen Watsin Genetech Ltd. Dibasic sodium phosphate was purchased from Chengdu Jinshan Chemical Reagent Co., Ltd. Potassium dihydrogen phosphate was purchased from Sangon Biotech (Shanghai) Co., Ltd. Nutrient agar and eosin methylene blue were purchased from Beijing Solarbio Science and Technology Co., Ltd. Phosphoric acid, methanol, n-butanol, acetic anhydride, sulfuric acid, formaldehyde solution, sodium chloride, sodium citrate, and citric acid were purchased from Chengdu Kelong Chemical Reagent Factory (Chengdu, China). The diabetes-inducing agent streptozotocin (STZ) (S0130) was purchased from Sigma-Aldrich (Sigma-Aldrich, Shanghai, China).

### 2.2. Animals

Kunming (KM) mice (CV, female/male ratio = 1 : 1, 30 g ± 5 g) were supplied by the Third Military Medical University Daping Hospital (License number: SCXK Yu 2012–0005) and housed in the animal room of the School of Pharmacy, Zunyi Medical University. Mice living conditions were ventilation at room temperature of 25 ± 2°C, quiet environment, natural lighting day and night, and free access to water and food.

Sprague Dawley (SD) rats (SPF, female/male ratio = 1 : 1, 250 g ± 20 g) were supplied by the Third Military Medical University Daping Hospital (License number: SCXK Yu 2012–0005) and housed in the SPF animal room of the Department of Pharmacology, Zunyi Medical University. Rat living conditions were ventilation at room temperature of 22 ± 2°C, quiet environment, natural lighting day and night, and free access to water and food.

Rabbits (CV, female/male ratio = 1 : 1, 2.5 kg ± 0.5 kg) were supplied by the Animal Experiment Center of Zunyi Medical University.

*Cavia porcellus* (CV, female/male ratio = 1 : 1, 300 g ± 30 g) were supplied by the Animal Experiment Center of Zunyi Medical College.

Rabbits and *Cavia porcellus* were housed in the animal room of the School of Pharmacy, Zunyi Medical University, with ventilation at room temperature of 25 ± 2° C, quiet environment, natural lighting day and night, and free access to water and food.

The animal experiment protocol was approved by the Institutional Animal Care and Use Committee of Zunyi Medical University (approval number: ZMU 2019-2-013).

### 2.3. Selection of Gel Adjuvants

#### 2.3.1. Selection of Matrix

The gel is oily and aqueous, widely used in clinical practice, easy to apply, and free of greasy feeling. The aqueous gel matrix is composed of polymer materials, mainly including carbomer, sodium alginate, chitosan, cellulose derivatives, starch, yarrow gum, and gelatin. The gel matrix was screened according to the characteristics and administration route of CATGNOG.

#### 2.3.2. Selection of Moisturizer

The high hydration degree of gel is conducive to the transdermal absorption of drugs, but in the storage process, the moisture content of gel is easy to be lost, resulting in the damage of stability and poor skin followability, so moisturizers are added to the gel matrix to solve the problem. Commonly used humectants are glycerol, propylene glycol, sorbitol, and polyethylene glycol. Dissolve each humectant in the aqueous solution at the ratio of 1 : 1, evenly spread it on the weighed glass slide, about 10 mg/cm 2, put it in an incubator at 37°C for 4 hours and then take it out, and calculate the moisture retention rate. The lower the moisture retention rate, the better the moisture retention effect.

(1) $$\begin{array}{c} \text{Moisture retention rate}\left(\%\right)=\frac{\left(M1\;-\;M2\right)}{\left(M1\;-\;M\right)}\times 100\%. \end{array}$$

Here, M (g) is the weight of the slide; M1 (g) is the mass before placing; M2 (g) is the mass after placing.

#### 2.3.3. Study on the Forming Time of Gel

The formability of 2% hydroxyethyl cellulose was studied. At room temperature and stirring speed of 380 r/min, the effects of overnight swelling of gel and different stirring times on gel appearance, coating extensibility, centrifugal stability, thermal storage stability, and freeze-thaw stability were studied, and the optimum forming time of gel was selected.

### 2.4. Study on Gel Preparations

#### 2.4.1. Comprehensive Evaluation Criteria for Gel

According to the comprehensive scoring standard of the sensory index and experimental gel index and the retention time of NO release greater than 2000 ppm as an index, the prescription of NO gel was screened. Total score = (5.0/NO NO release time) × NO release time + (5.0/maximum score of gel sensory and experimental index) × gel sensory and experimental index score, with gel appearance characteristics, uniformity, coating, centrifugal stability, thermal storage stability, cold storage stability, and freeze-thaw stability as the scoring criteria of the sensory and experimental index, as shown in Table 1.

#### 2.4.2. Preparation of Gel

The gel is composed of gel 1 and gel 2 and is prepared according to the following steps.

*(i) Gel 1*. (1) *Centella asiatica* total glycosides (purity: 47.95%) were dissolved with an appropriate amount of water. (2) After complete dissolution of ascorbic acid with an appropriate amount of water, add glycerol and stir well. (3) Add (1) into (2), stir well, add hydroxyethyl cellulose, and stir at 380 r/min for 2 h. (4) Add ethylparaben (dissolve with ethanol; the ratio of ethylparaben to ethanol is 1 : 4) and azone, stir well, and add distilled water to constant volume to 100 ml.

*(ii) Gel 2*. (1) Sodium nitrite was completely dissolved with an appropriate amount of water, and glycerol was added and stirred evenly. (2) Add hydroxyethyl cellulose and stir at 380 r/min for 2 h. (3) Add g of ethylparaben (dissolve with ethanol; the ratio of ethylparaben to ethanol is 1 : 4) and of azone, stir thoroughly, and add distilled water to make 100 ml.

### 2.5. Single-Factor Test

1. Investigation of active ingredient content
2. Five pieces of *Centella asiatica* total glycosides were accurately weighed, which were 4 g, 8 g, 12 g, 16 g, and 20 g, and hydroxyethyl cellulose 1 g, azone 1 g, and glycerol 10 g were also accurately weighed. The gel agent was prepared in accordance with the method described in 2.5.2, and the content of *Centella asiatica* total glycosides was selected by the comprehensive scoring criteria of the gel agent.
3. Investigation of hydroxyethyl cellulose content
4. Accurately weigh 1.6 g, 2.0 g, 2.4 g, 2.8 g, and 3.2 g of hydroxyethyl cellulose, 12 g of *Centella asiatica* total glycosides, 1 g of azone, and 10 g of glycerol, prepare the gel according to the method described in 2.5.2, and screen the content of hydroxyethyl cellulose by the comprehensive scoring standard of gel.
5. Investigation of azone content
6. Accurately weigh 1.0 g, 2.0 g, 3.0 g, 4.0 g, and 5.0 g of azone, 12 g of *Centella asiatica* total glycosides, 2.4 g of hydroxyethyl cellulose, and 10 g of glycerol, prepare the gel according to the method described in 2.5.2, and screen out the content of azone according to the comprehensive scoring criteria for gel.
7. Investigation of glycerol content
8. Accurately weigh 0.0 g, 2.5 g, 5.0 g, 7.5 g, and 10.0 g of glycerol, 12 g of *Centella asiatica* total glycosides, 2.4 g of hydroxyethyl cellulose, and 2 g of azone, prepare the gel according to the method described in 2.5.2, and select the glycerol content according to the comprehensive scoring standard of gel.

### 2.6. Optimization of CATGNOG Prescription by Orthogonal Test

Through the analysis of single-factor investigation results of the gel formation process, the L_9_(3^4^) orthogonal test was carried out with the *Centella asiatica* total glycosides (A), hydroxyethyl cellulose (B), azone (C), and glycerol (D) as four factors. The optimum process is determined by the score of the gel-forming index and NO release time, and the factor level is shown in Table 2.

### 2.7. Study on Percutaneous Permeability

#### 2.7.1. Establishment of HPLC Method for Asiaticoside and Madecassoside

1. Chromatographic conditions
2. The analysis was performed on an Agilent 1260 HPLC system (American Agilent Technologies Co., Ltd.): chromatographic column: ZORBAXSB-C 18 (250 × 4.6 mm, 5 *μ*m); mobile phase: acetonitrile (2 mmol/L): *β*-CD = 25 : 75 (add 0.1% glacial acetic acid as modifier); flow rate: 1.0 mL/min; detection wavelength: 205 nm; column temperature: 30°C; sample volume: 10 *μ*L.
3. Investigation of the linear relationship
4. Accurately weigh asiaticoside and madecassoside reference substance, and add a proper amount of methanol to prepare a stock solution containing 4000 *μ*g of asiaticoside and 4000 *μ*g of madecassoside per milliliter. Pipet the appropriate stock solution, add methanol, and shake well to prepare the mixed reference solution containing asiaticoside at concentrations of 20, 40, 80, 160, 320, 640, and 1280 *μ*g/mL and madecassoside at concentrations of 30, 60, 120, 240, 480, 960, and 1920 *μ*g/mL, respectively. Measure under the above chromatographic conditions, and record the peak area. The regression equation was obtained by taking the mass concentration of the mixed reference substance as the *x*-axis and the peak area as the *y*-axis.
5. Particularity study
6. Draw 10 *μ*L of the mixed reference solution, test solution, and negative reference solution, respectively, inject them into the high-performance liquid chromatograph (HPLC, Agilent 1260, Agilent, Palo Alto, CA, USA), and record the chromatogram.
7. Precision study
8. Take the same mixed reference solution, continuously inject it six times within the same day, measure it under the proposed chromatographic conditions, record the peak area, and calculate the relative standard deviation.
9. Repeatability study
10. Take six copies of the same gel, prepare the gel solution according to the method, determine the content under the proposed chromatographic conditions, and record the peak areas.
11. Stability study
12. Take a proper amount of the same solution, determine the content at 0, 2, 4, 6, 8, and 24 h under the above chromatographic conditions, and record the peak area.
13. Spike-and-recovery experience
14. Take 0.5 g of gel 1 and gel 2, respectively, stir well, fully react for 5 min, add 25 ml of methanol, sonicate for 10 min, and filter with 0.45 *μ*m microporous filter membrane to obtain CATGNOG background solution. Accurately weigh the asiaticoside and madecassoside reference substances, and add methanol for ultrasonic dissolution to prepare a mixed control solution containing 0.10 mg asiaticoside and 3.43 mg madecassoside per milliliter. Accurately transfer 100 *μ*L of background solution into three sampling bottles, and add 80, 100, and 120 *μ*L of standard solution, respectively, to prepare low, medium, and high concentration test solutions in triplicate for content determination under the proposed chromatographic conditions.

#### 2.7.2. Preparation of Skin

1. Preparation of intact skin
2. The abdominal hair of KM mice was shaved with an electric hair clipper, and then they were depilated with depilatory cream and wiped clean with normal saline. The mice were sacrificed by cervical dislocation followed by decapitation, and the depilated skin of the abdomen was dissected surgically. Carefully scrape off subcutaneous fat and mucous membrane, wash it repeatedly with normal saline and suck it dry with filter paper, cut it into circles of appropriate size, spread it on the filter paper, and put it at −4°C for standby the next day.
3. Preparation of exfoliated skin
4. Peel the stratum corneum with transparent tape by even force and parallel operation more than 25 times until the tape is free of dirt as the standard for completely peeling the stratum corneum. Cut into a circle with appropriate size, lay flat on the filter paper, and put it at −4°C for standby the next day.

### 2.8. *In Vitro* Transdermal Test

The prepared skin was fixed in the diffusion pool (the volume of receiving pool was 8 mL) (Kaikai Technology Trading Co., Ltd., Shanghai, China), with the cuticle upward and the dermal layer in complete contact with the receiving solution. The effective permeation area is 3.46 cm^2^, and the receiving solution is normal saline. The circulation temperature of the diffusion tank is controlled at 37 ± 0.5°C, and the constant speed of magnetic stirring in the receiving tank is 300 r/min.

After the whole circulatory system reaches the set temperature, the skin is balanced in the diffusion pool for 0.5 h, the fresh receiving solution at the same temperature and volume is replaced, and the bubbles are discharged, so that the skin is completely contacted with the dermis. Add 1 g of gelling agent, stir well, sample 5 mL from each receiving pool by syringe at 3, 6, 9, 12, 15, and 24 h, and then immediately add normal saline of the same temperature and volume. A total of 15 ml of the receiving solution from three receiving pools was concentrated to a constant weight with dry extract, dissolved with 1 ml of methanol, filtered with 0.45 mm microporous membrane, and injected according to the above chromatography method. Each assay was performed three times, and the results were averaged.

### 2.9. Calculation of Accumulative Permeability per Unit Area of *Centella asiatica* Total Glycosides

Calculate the accumulative permeability per unit area (*Q*_*n*_) according to the following formula:

(2) $$\begin{array}{c} Q_{n}=\frac{C_{n}V\;+\;\sum_{i=1}^{n-1}C_{i}V_{i}}{A}, \end{array}$$

where *C*_*n*_ is the drug concentration at the nth sampling point (*μ*g/mL); *V* is the receiving liquid volume (mL); *C*_*i*_ is the drug concentration at the ith sampling point (*μ*g/mL); *V*_*i*_ is the sampling volume (mL); *A* is the penetration area (3.46 cm^2^).

### 2.10. Analysis of Drug Release Characteristics and Pharmacokinetic Model

Commonly used *in vitro* drug release data fitting kinetic models include zero-order (*Q* = a+bt) and Higuchi (*Q* = a+bt^1/2^) kinetics models. Zero-order and Higuchi kinetics models were fitted to the cumulative release per unit area of CATGNOG against time. The obtained slope *J* is the transdermal rate constant, and EF = *J*_(intact skin)_/*J*_(exfoliated skin)_ is used to evaluate the permeability of the stratum corneum to the drug [39].

### 2.11. Physicochemical Properties of CATGNOG

According to the 2015 edition of China Pharmacopoeia (four), the quality inspection is carried out under the requirements of the gel.

### 2.12. Appearance

Gel should be uniform and delicate and dry or liquefied and kept gel-like. Under these conditions, gel-related inspection should be carried out.

### 2.13. pH Value

Weigh 1 g of gel 1 and 1 g of gel 2, respectively, put them in a beaker, add 25 mL of distilled water for dilution, and fully stir and disperse them. After ultrasonic treatment for 10 minutes, the pH value was measured with a pH meter (Sartorius Scientific Instrument Co., Ltd., Beijing, China).

### 2.14. Viscosity

An appropriate amount of the gel was loaded into a centrifuge tube, the appropriate rotor and rotation speed were selected, and its viscosity was measured using a viscometer (Changji Geological Instrument Co., Ltd., Shanghai, China).

### 2.15. Determination of Active Ingredient in CATGNOG

#### 2.15.1. Determination of *Centella asiatica* Total Glycosides in CATGNOG

Take three batches of CATGNOG, take 0.5 g of gel 1 and gel 2, respectively, stir well, react fully for 5 min, add 25 ML of methanol, subject to ultrasonic for 10 min, filter with microporous membrane to obtain CATGNOG test solution, and determine the content by HPLC.

#### 2.15.2. Determination of NO in CATGNOG

Weigh 0.3 g each of gel 1 and gel 2, quickly mix well and place them in a 10 L vacuum container, and determine the content of NO in the container with a NO gas analyzer (Skesen Gas Detection Equipment Co., Ltd., Guangdong, China).

### 2.16. Stability Study

#### 2.16.1. Centrifugation Test

1. Low-speed centrifugation test
2. Take three batches of gels with different batches, respectively, 5 g of gel 1 and gel 2, put them into a centrifuge tube, and centrifuge at 3000 r/min for 30 minutes to observe the appearance and coating extensibility.
3. High-speed centrifugation test
4. Take three batches of gels with different batches, respectively, 5 g of gel 1 and gel 2, put them into a centrifuge tube, and centrifuge at 12000 r/min for 30 minutes to observe the appearance and coating extensibility.

#### 2.16.2. High-Temperature Test

Take three batches of gels with different batches, respectively, 5 g of gel 1 and gel 2, place them in centrifuge tubes, wrap the centrifuge tubes with tin paper to protect from light, seal them and place them in a drying oven with a temperature of 60 ± 2°C, take samples at 0, 5, and 10 d, observe their appearance, determine the viscosity and the content of *Centella asiatica* total glycosides, and compare with the results on day 0.

#### 2.16.3. Short-Time Test

Take three batches of gels with different batches, respectively, 5 g of gel 1 and gel 2, place them in centrifuge tubes, wrap the centrifuge tubes with tin paper to protect from light, seal them and place them in an environment with a temperature of 25 ± 5°C and relative humidity of 60 ± 10°C, take samples at the 1st month, 2nd month, 3rd month, and 6th month, observe their appearance, determine the viscosity and the content of *Centella asiatica* total glycosides, and compare with the results on day 0.

#### 2.16.4. Low-Temperature Test

Take three batches of gels with different batches, respectively, 5 g of gel 1 and gel 2, place them in centrifuge tubes, wrap the centrifuge tubes with tin paper to protect from light, seal them and place them in an environment with a temperature of 4 ± 2°C, take samples at 0, 5, 10, 15, and 90 d, observe their appearance, determine the viscosity and the content of *Centella asiatica* total glycosides, and compare with the results on day 0.

### 2.17. Study on Acute Dermal Toxicity and Reproductive Toxicity

#### 2.17.1. Preparation of Test Rat Skin

1. Intact skin
2. Thirty-two SD rats were adaptively fed in a single cage for three days, and the hair on both sides of the back spine was removed by electric push scissors, with a depilation area of 5 × 6 cm. 24 hours before administration, check whether the skin is damaged. The breakage skin was not suitable for the skin integrity test.
3. Breakage skin
4. In 32 SD rats, the depilated area was disinfected with ethanol after depilation, and then a scratch marked with # was made on the depilation area of rats with an ethanol sterilized scalpel, taking the blood oozing from the epidermis as the standard.

#### 2.17.2. Grouping and Administration

SD rats were divided into eight groups, with four rats in each group, male and female half. The intact skin group and breakage skin group was divided into four groups: blank matrix group and gel low-, medium-, and high-dose groups (1 g, 2 g, and 4 g). The medicine was wrapped with two layers of gauze and fixed with nonirritating adhesive tape. After 24 hours after administration, the test drug was taken out, and the administration site was cleaned with warm water. One hour of drug removal was the first observation point for seven days.

#### 2.17.3. Observation Indicators

The body weight change, skin hair color, eye and mucosa change, respiration, central nervous system, limb activity, poisoning, and other manifestations were observed and recorded daily. In case of death, macroscopic observation and autopsy should be performed in time. In case of any visible lesion, HE should be performed. At the same time, blood routine tests were performed, such as total red blood cell (RBC), total white blood cell (WBC), hemoglobin (HGB), and total platelet (PLT). Seven days later, the uterus and ovaries of female rats, testicles, and epididymis of male rats in each group were taken, and the surrounding fat and connective tissue were removed as much as possible with scissors and eye pliers. The separated organs were placed in normal saline and washed and dried with filter paper, and the organs' wet weight was calculated. The viscera index was calculated by the following formula:

(3) $$\begin{array}{c} \text{Viscera index }\left(\%\right)=\frac{\text{Organ weight}}{\text{body weight}}\times 100\%. \end{array}$$

Histopathological evaluation (HE) staining: the tissues of reproductive organs were fixed in 10% neutral buffered formalin, and after fixation, routine paraffin-embedded sections (4 *μ*m) were examined pathologically. HE staining was performed to observe histopathological changes under a 200-fold electron microscope.

### 2.18. Study on Skin Irritation and Reproductive Toxicity

#### 2.18.1. Preparation of Test Rabbits

1. Intact skin
2. Sixteen healthy rabbits were adaptively fed in a single cage for three days, and the hair on both sides of the back spine was removed by electric push scissors, with a depilation area of 5 × 6 cm. 24 hours before administration, check whether the skin is damaged. The breakage skin was not suitable for the skin integrity test.
3. Breakage skin
4. In 16 healthy rabbits, the depilated area was disinfected with ethanol after depilation, and then a scratch marked with # was made on the depilation area of rabbits with an ethanol sterilized scalpel, taking the blood oozing from the epidermis as the standard.

#### 2.18.2. Grouping and Administration

Rabbits were divided into 4 groups, with 4 rats in each group, male and female half. The intact and breakage skin groups were divided into two groups: blank matrix and CATGNOG groups. Weigh 1 g of the test drug, evenly smear it on the left side of the alopecia area, blank smear gel on the right side, cover it with two layers of gauze, and fix it with nonirritating tape. After 24 hours after application, remove the test drug and wash the administration site with warm water. At 1, 24, 48, and 72 hours after removal of the residual drug, the injection sites were observed macroscopically for redness, swelling, and erythema.

#### 2.18.3. Observation Indicators

The skin irritation response was evaluated according to the skin irritation response scoring criteria and evaluation criteria, with the highest total score of 8 points (Tables 3 and 4), and a blood routine examination was performed at the same time. After 3 days, the viscera index was calculated, and HE staining was performed in the reproductive organs. Rabbit skin irritation was scored at 1, 24, 48, and 72 hours after administration.

### 2.19. Skin Sensitization Test

#### 2.19.1. Preparation of Test *Cavia porcellus*

Thirty *Cavia porcellus* (female/male ratio = 1 : 1) were adaptively fed in a single cage for 3 days, and the hair on both sides of the back spine was removed by electric push scissors, with a depilation area of 3 × 3 cm. 24 hours before administration, check whether the skin is damaged. The breakage skin was not suitable for the skin integrity test.

#### 2.19.2. Grouping and Administration

*Cavia porcellus* were divided into 3 groups, with 10 *Cavia porcellus* in each group, which were blank matrix group, CATGNOG group, and positive control group (1% 2,4-dinitrochlorobenzene).

1. Induction exposure
2. Take 0.2 g of the drug to be tested, apply it to the depilation area on the left side of the animal, cover it with two layers of gauze, and fix it with nonirritating adhesive plaster to make the drug in continuous contact with the skin for 6 h. The procedure was repeated once on the 7th and 14th days, respectively.
3. Eliciting exposure
4. On the 14th day after sensitization, 0.2 g of the test drug was applied to the hair removal area on the right side of *Cavia porcellus*, and the positive control was replaced with 0.1% 2, 4-dinitrochlorobenzene. After 6 hours, the test drugs were removed. They were observed immediately, and skin allergy was observed again at 24, 48, and 72 hours.

#### 2.19.3. Observation Indicators

The score results were evaluated according to the evaluation standard of skin sensitization (Table 5), and a blood routine was carried out at the same time. After three days, the uterus, ovaries, testes, and epididymis of each *Cavia porcellus* were taken out, HE staining was performed, and the viscera index was calculated. 6 h after administration, observe the skin allergy of the *Cavia porcellus*.

### 2.20. Study on Pharmacodynamics of CATGNOG Promoting Wound Healing

#### 2.20.1. Experimental Design

After feeding the rats for one week, they were randomly divided into two groups: normal groups and DM groups. Each group was randomly divided into 6 groups: model group, blank group, high-, medium-, and low-dose groups of CATGNOG (16%, 8%, and 4%), and positive control group. Each group was divided into 3-, 7-, 10-, and 14-day groups, with 7 rats in each group.

1. Model group.
2. Blank control (blank matrix) group.
3. Low-dose (4% *Centella asiatica* total glycosides) group.
4. Medium-dose (8% *Centella asiatica* total glycosides) group.
5. High-dose (16% *Centella asiatica* total glycosides) group.
6. Positive control (recombinant human epidermal growth factor solution, LST) group.

Rats in the diabetic group were injected intraperitoneally with STZ at the dose of 50 mg/kg. 72 hours later, blood samples were taken from the tail vein to measure blood glucose. If the concentration of blood glucose was ≥16.7 mmol/L, the diabetic model was established successfully. The diabetic rats were monitored, and the full-thickness skin defect model was made after confirming the obvious characteristics of diabetes. Rats were surgically cut in the back to create a round wound of about 1.8 cm, deep in subcutaneous. Rats were naturally exposed on day 1, and day 2 was counted as the first day of the experiment. Local administration was carried out every morning and evening, with the drug covering the wound as the standard. The wounds were washed with normal saline regularly, and the rats in the model group were only washed with normal saline without drug intervention. The rats in each group were sacrificed on days 3, 7, 10, and 14.

#### 2.20.2. Bacterial Count and Identification

Bacterial sampling was taken from the wound of rats on days 3 and 7, and the whole process was operated under sterile environment. The sterilized 0.5 cm^2^ square filter paper was infiltrated with sterile saline abutted to the wound surface. 10 seconds later, the filter paper was put into 3 ml of sterile saline, and parallel experiments were conducted 3 times. After shaking well, 200 *μ*L was evenly inoculated on nutrient agar medium and eosin methylene blue medium, respectively. After incubation at 37°C for 24 hours, count and identify the bacteria on the culture medium.

#### 2.20.3. Wound Healing Rate

The ulcer wounds of rats were photographed on the morning of the 1st, 3rd, 7th, 10th, and 14th days, and the images were processed using Adobe Photoshop CS5 software to calculate the wound area. The wound healing rate is calculated according to the following formula:

(4) $$\begin{array}{c} \text{wound healing rate}=\frac{\text{Original wound area}-\text{Unhealed wound area}}{\text{Original wound area}}\times 100\%. \end{array}$$

#### 2.20.4. Histopathological Evaluation (HE) Staining

On the 14th day, the skin tissue of the wound was fixed in 10% neutral formalin buffer, and then the pathological routine paraffin-embedded sections (4 *μ*m) were prepared, which were stained with hematoxylin eosin. OLYMPUS BX43 microscope was used to observe the histopathological morphology, granulation tissue proliferation, and epithelial tissue formation.

### 2.21. Statistical Analysis

The experimental results were presented as $\bar{X}\pm s$. Student's *t*-test was used to compare groups of two. For more than two groups, a one-way ANOVA was used to evaluate statistical significance (*P* < 0.05 and *P* < 0.01). GraphPad Prism Version 7.00 for Windows (GraphPad Software, La Jolla California USA) was used to perform statistical analysis.

## 3. Results

### 3.1. Selection of Gel Adjuvants

#### 3.1.1. Selection of Matrix

*Centella asiatica* total glycosides nitric oxide gel (CATGNOG) is composed of acid agent and nitrogen agent. NO must be generated in an acidic environment, while carbopol must be in a neutral environment to become a gel. Considering all kinds of factors, hydroxyethyl cellulose, whose viscosity and surface tension are rarely affected by additives, is selected as the gelling agent matrix.

#### 3.1.2. Selection of Moisturizer

Humectant not only promotes wound healing but also maintains specific stability in gel storage. The results showed that the moisture retention rates of glycerin, propylene glycol, and polyethylene glycol were 32.30%, 44.39%, and 45.24%, respectively. The moisturizing effect of glycerol was better than that of propylene glycol and polyethylene glycol. Therefore, glycerol was selected as the humectant of the gel. The results are summarized in Table 6.

#### 3.1.3. Study on the Forming Time of Gel

Through comparison, the forming condition of hydroxyethyl cellulose was determined as 380 r/min and stirring for 2 hours. The gel prepared under these conditions has the advantages of uniform spreading, delicate texture, easy application, and good stability. The results are shown in Table 7.

### 3.2. Study on Gel Preparations

#### 3.2.1. Single-Factor Test

**Figure 1:**
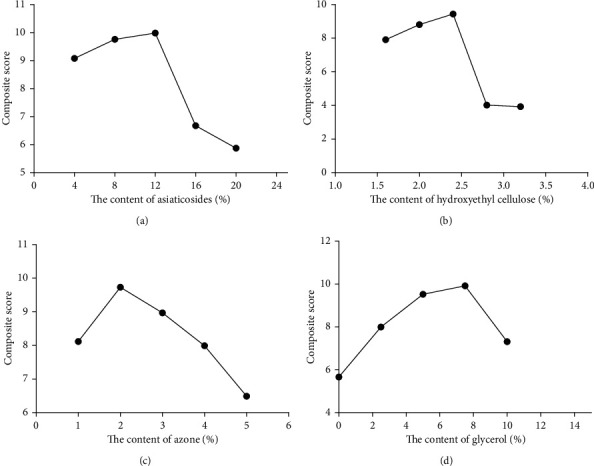
The test of single factor. (a) The content of *Centella asiatica* total glycosides. (b) The content of hydroxyethyl cellulose. (c) The content of azone. (d) The content of glycerol.

*(i) Investigation of Active Ingredient Content*. It can be seen from Figure 1(a) that the total score is the highest when the content of *Centella asiatica* total glycosides is 12%. Therefore, the content of *Centella asiatica* total glycosides was 8%, 12%, and 16%.

*(ii) Investigation of Hydroxyethyl Cellulose Content*. It can be seen from Figure 1(b) that the total score is the highest when the content of hydroxyethyl cellulose is 2.4%. Therefore, the content of hydroxyethyl cellulose was 2.0%, 2.4%, and 2.8%.

*(iii) Investigation of Azone Content*. It can be seen from Figure 1(c) that the total score is the highest when the content of an azone is 2.0%. Therefore, the content of azone was 1.0%, 2.0%, and 3.0%.

*(iv) Investigation of Glycerol Content*. It can be seen from Figure 1(d) that the total score is the highest when the content of glycerol is 7.5%. Therefore, the content of glycerol was 5.0%, 7.5%, and 10.0%.

#### 3.2.2. Optimization of CATGNOG Prescription by Orthogonal Test

There are many evaluation indexes for gel. In order to prevent a single evaluation indicator from causing partial results, this experiment selects sensory indicators, experimental indicators, and comprehensive experimental indicators for the release time of NO to make a comprehensive evaluation of the gels and select the best prescription. The results of the orthogonal test are given in Table 8. Prescriptions 1 and 2 are homogeneous and easy to coat, and the release time of nitric oxide is relatively slow.

### 3.3. Study on Percutaneous Permeability

#### 3.3.1. Establishment of HPLC Method for Asiaticoside and Madecassoside

**Figure 2:**
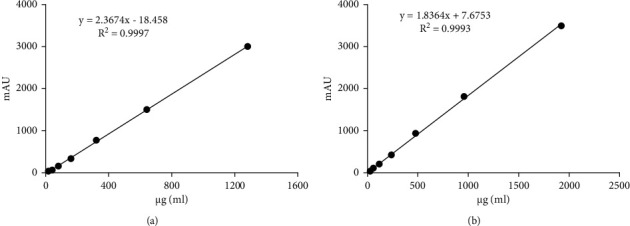
Standard curve. (a) Asiaticoside; (b) madecassoside.

*(i) Investigation of Linear Relationship*. Regression equation of asiaticoside is *y* = 2.3674x-18.458 (*r* = 0.9997; *n* = 7); regression equation of madecassoside is *y* = 1.8364*x* + 7.6753 (*r* = 0.9993; *n* = 7), as shown in Figure 2. The results showed that there was a good linear relationship between asiaticoside and its peak area in the mass range of 20 ∼ 1280 *μ*g/ml and madecassoside in the mass range of 30 ∼ 1920 *μ*g/mL.

**Figure 3:**
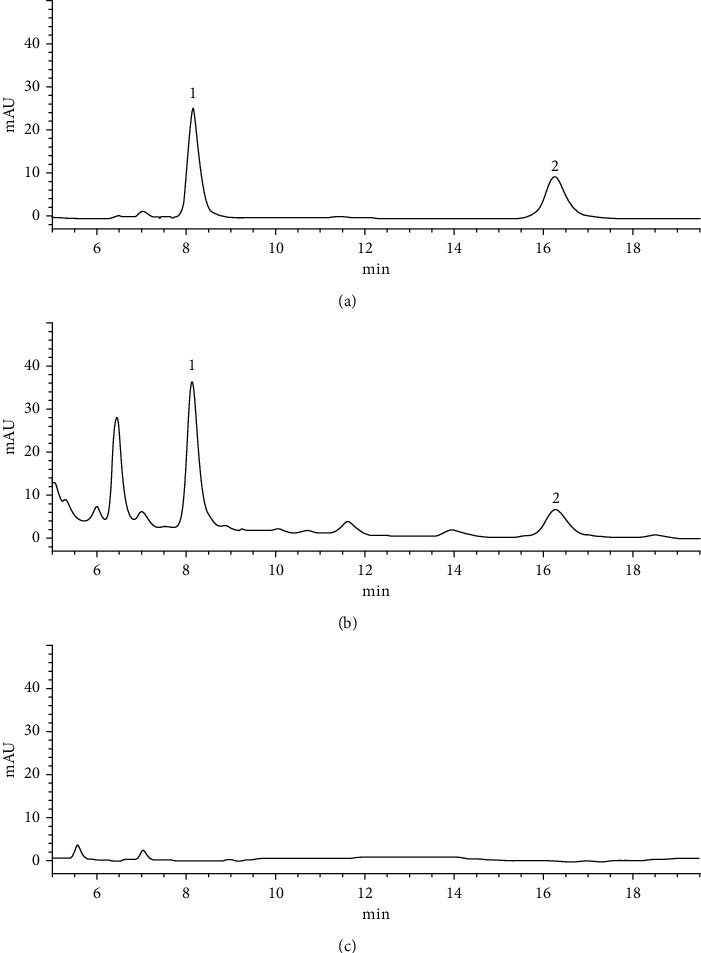
The HPLC chromatogram. (a) Reference solution. (b) Test solution. (c) Negative reference solution: (1) madecassoside; (2) asiaticoside.

*(ii) Particularity Study*. The negative control solution had no significant chromatographic peaks at the corresponding positions of asiaticoside and madecassoside, which indicates that the negative control had no interference and specificity to the determination, as shown in Figure 3.

*(iii) Precision Study*. The RSD of asiaticoside and madecassoside was 0.67% and 1.57%, respectively, which indicates that the precision of the instrument was good. The results are presented in Table 9.

*(iv) Repeatability Study*. The RSD of asiaticoside and madecassoside was 1.51% and 2.94%, respectively, which indicates that the method has good repeatability. The results are shown in Table 10.

*(v) Stability Study*. The RSD of asiaticoside and madecassoside was 3.57% and 2.53%, respectively, which indicates that the test solution was basically stable within 24 h. The results are available in Table 11.

*(vi) Spike-and-Recovery Experience*. The average recoveries rates of asiaticoside and madecassoside were 97.33% and 98.44%, and the RSD was 0.75% and 2.00%, respectively. The results showed that the RSD was less than 5%, which indicated that asiaticoside and madecassoside met the accuracy requirements of the sample recovery rate. The results are imputed in Tables 12 and 13.

#### 3.3.2. Transdermal Testing *In Vitro*

**Figure 4:**
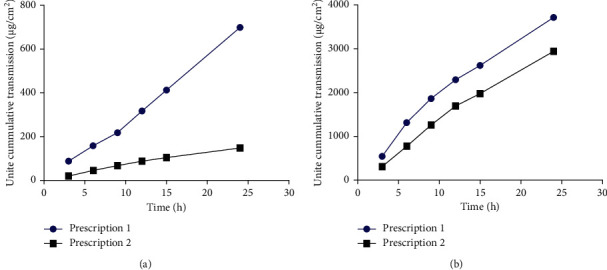
The *in vitro* release curve of *Centella asiatica* total glycosides. (a) Intact skin; (b) exfoliated skin.

In the process of treating the skin of mice, it is necessary to remove the fat and mucosal tissue and remove the subcutaneous blood vessels. Because the gel will penetrate the skin and enter blood vessels, affecting the experimental results, the subcutaneous tissue of abdominal skin is less than that of back skin. Therefore, the abdominal skin is selected as the carrier of transdermal absorption *in vitro*. After the skin is prepared, put it in the refrigerator for later use. Time should not be too long because the storage time will affect the transdermal absorption of the drug. The average temperature of the human surface is 33.5°C. In order to avoid excessive deviation of experimental results, the temperature of the diffusion cell is 37.0°C, which is because the rat skin is in the state and the activity is low. The increase in temperature helps to improve the activity. From the *in vitro* release of prescription 1 and prescription 2, the cumulative release per unit area of *Centella asiatica* total glycosides from intact and exfoliated skin was higher in prescription 1 than prescription 2 at each point. The results are provided in Table 14 and Figure 4. Therefore, we choose prescription 1 with a complete drug release as the optimal prescription. Gel 1 is composed of 8 g of *Centella asiatica* total glycosides (purity: 47.95%), 8 g of acid, 2 g of hydroxyethylcellulose, 1 g of azone, 5 g of glycerol, and 0.2 g of ethylparaben. Gel 2 is composed of 2 g of alkaline, 2 g of hydroxyethylcellulose, 1 g of azone, 5 g of glycerol, and 0.2 g of ethylparaben.

#### 3.3.3. Analysis of Drug Release Characteristics and Pharmacokinetic Model

It can be seen from Table 15 that the release rate of the gel acting on the decorticated skin is greater than that on the intact skin.

### 3.4. Physicochemical Properties of CATGNOG

#### 3.4.1. Appearance

The gel paste is uniform and delicate and has good coating properties. CATGNOG is composed of gel 1 and gel 2. Gel 1 contains *Centella asiatica* total glycosides, so gel 1 is brownish-yellow translucent semisolid, and gel 2 is colorless transparent semisolid.

#### 3.4.2. pH Value

The pH value of the gel meets the requirements, which is 4.67. The results are reported in Table 16.

#### 3.4.3. Viscosity

Viscosity was measured by NDJ-1 A rotary viscometer, and the results showed that the viscosity of CATGNOG was between 10 and 20 Pa.s.

### 3.5. Determination of Active Ingredient in CATGNOG

#### 3.5.1. Determination of *Centella asiatica* Total Glycosides in CATGNOG

The average content of the main drug in the three batches of CATGNOG is 19.49 mg/g, and the RSD is 1.18%. The results are presented in Table 17.

#### 3.5.2. Determination of NO in CATGNOG

**Figure 5:**
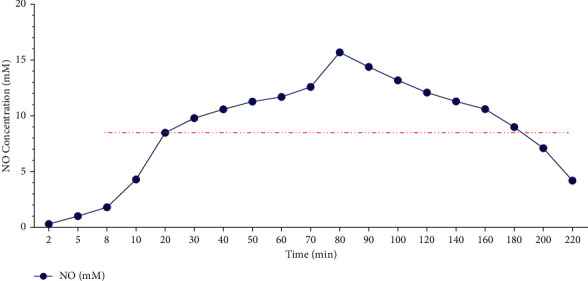
NO release curve *in vitro* of CATGNOG.

The effective concentration of NO released in the gel was maintained for nearly 3 h, which indicated that NO in the gel had a slow-release effect, and the results are shown in Figure 5.

### 3.6. Stability Study

#### 3.6.1. Centrifugation Test

*(i) Low-Speed Centrifugation Test*. After centrifugation, gel 1 and gel 2 are uniforms and transparent. Gel 1 is brownish-yellow, and gel 2 is colorless and transparent semisolid, without layering, with good spreading and uniform and delicate coating.

*(ii) High-Speed Centrifugation Test*. After centrifugation, gel 1 and gel 2 are uniform and transparent. Gel 1 is brownish-yellow, and gel 2 is colorless and transparent semisolid, without layering, with good spreading and uniform and delicate coating.

#### 3.6.2. High-Temperature Test

After five days under high temperature, the color of gel deepened, the spreadability became worse, the viscosity decreased sharply, and the *Centella asiatica* total glycosides content reduced. This gel is not suitable for high-temperature storage. Results are summarized in Table 18.

#### 3.6.3. Short-Time Test

In the accelerated test, the color of gel 1 deepened, and spreadability decreased at 30 days, while the viscosity of gel 2 changed significantly, and the content of gel 2 did not change. Therefore, the gel is unstable under conditions of 25°C and 60% humidity. It is recommended to keep it at a low temperature. The results of the primary outcomes are shown in Table 19.

#### 3.6.4. Low-Temperature Test

In the low-temperature test, the appearance of the gel did not change, the coating was better, and the viscosity and *Centella asiatica* total glycosides content did not change significantly, which indicated that the gel has good stability. Therefore, it is suggested that the gel should be stored at a low temperature, which is 4 ∼ 8°C. The results are detailed in Table 20.

#### 3.6.5. Study on Skin Irritation and Reproductive Toxicity

CATGNOG is a kind of skin preparation for external application. In order to study its skin safety, it is necessary to conduct skin toxicity tests. At present, rats and rabbits are often taken as research objects in skin toxicity tests, and the drug safety of the preparation can be judged by observing the toxic reaction of animals after topical application.

#### 3.6.6. The effect of CATGNOG on the weight change of rat

There was no change in daily activities and no significant difference in weight before and after administration (*P* > 0.05). The results are tabulated in Table 21.

#### 3.6.7. The Effect of CATGNOG on Blood Routine of Rat

There was no abnormal image in blood routine examination, which was not statistically significant compared with the blank group (*P* > 0.05). The results are depicted in Table 22.

#### 3.6.8. The Effect of CATGNOG on the Viscera Index of Rat

The viscera results of rats are detailed in Table 23, which were not statistically significant compared with the control group (*P* > 0.05).

#### 3.6.9. Histopathological Examination (HE) of Rat Reproductive Organs

**Figure 6:**
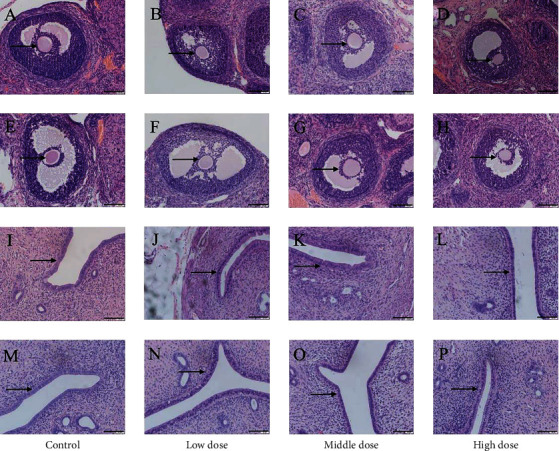
Effects of CATGNOG on the reproductive organs of female rats (H&E, ×200). (A–H) Ovary. (I–P) Uterus. (A–D, I–L) Intact skin. (E-F, M–P) Breakage skin. Scale bar: 50 *μ*m.

The HE of reproductive organs of female rats revealed that the ovarian tissue structure of the blank control group was intact, the follicle volume was large, the fluid content in the follicular cavity was high (shown by black arrows), and the corpus luteum and white bodies developed normally (Figures 6(a) and 6(e)). Compared with the blank control group, the ovaries of the low-, medium-, and high-dose groups had no obvious changes (Figures 6(b)–6(d) and 6(f)–6(h)). In the blank control group, the uterus has a standard shape, and the outer membrane, myometrium, and endometrium have precise contours. The epithelial cells of the uterine mucosa are monolayer columnar cells (shown by black arrows), and the glandular cavity is relatively large (Figures 6(i) and 6(m)). Compared with the blank control group, there were no significant changes in the uterus in the low-, medium-, and high-dose groups (Figures 6(i)–6(l) and 6(m)–6(p)).

**Figure 7:**
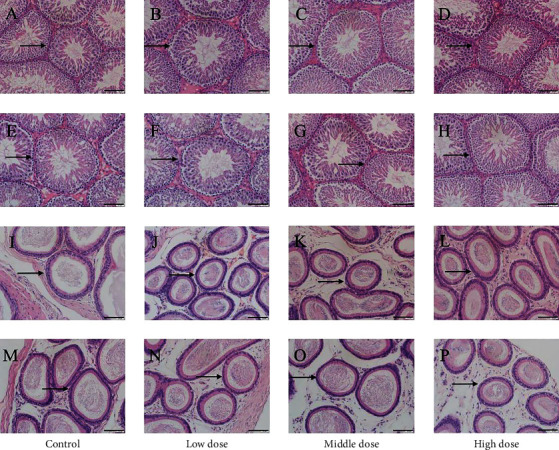
Effects of CATGNOG on the reproductive organs of male rats (H&E, ×200). (A–H) Testis. (I–P) Epididymis. (A–D, I–L) Intact skin. (E-F, M–P) Breakage skin. Scale bar: 50 *μ*m.

The HE of reproductive organs of male rats showed that the cells in testicular seminiferous tubules of the blank control group were closely arranged (shown by black arrows), and there were many spermatogenic cells at all levels (Figures 7(a) and 7(e)). Compared with the blank control group, the testis of the low-, medium-, and high-dose groups had no obvious changes (Figures 7(b)–7(d) and 7(f)–7(h)). In the blank control group, the efferent tubules of the epididymis are regular in shape, which are composed of single-layer high columnar cells with a large number of sperms in the lumen (Figures 7(i) and 7(m)). Compared with the blank control group, the epididymis had no noticeable changes at low, medium, and high doses (Figures 7(i)–7(l) and 7(m)–7(p)).

### 3.7. Study on Skin Irritation and Reproductive Toxicity

#### 3.7.1. Effects of CATGNOG on Rabbit Skin Irritation

The rabbit skin irritation was scored at 1 h, 24 h, 48 h, and 72 h after administration, and the scores were all lower than 0.5. The results are described in Table 24.

#### 3.7.2. The Effect of CATGNOG on Blood Routine in Rabbit

There was no abnormal image in blood routine examination, which was not statistically significant compared with the blank group (*P* > 0.05). The results are provided in Table 25.

#### 3.7.3. The Effect of CATGNOG on the Viscera Index of Rabbit

The viscera results of rabbits are listed in Table 26, which were not statistically significant compared with the control group (*P* > 0.05).

#### 3.7.4. HE of Rabbit Reproductive Organs

**Figure 8:**
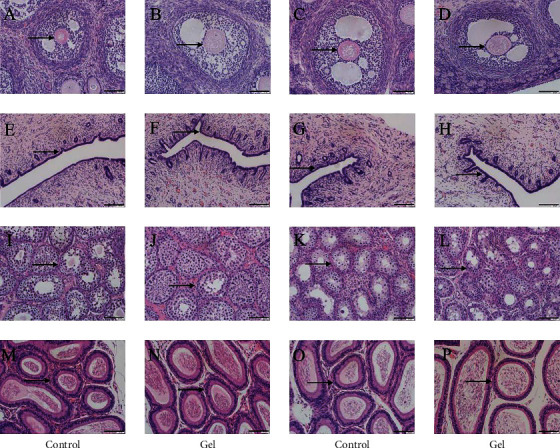
Effects of CATGNOG on the reproductive organs of rabbit (H&E, ×200). (A-D) Ovary. (E-H) Uterus. (I-L) Testis. (M-P) Epididymis. (A, B, E, F, I, J, M, N) Intact skin group. (C, D, G, H, K, L, O, P) Breakage skin group. Scale bar: 50 *μ*m.

HE of the reproductive organs of rabbits showed that the follicles in the blank control group were large, with many granular layers and abundant blood vessels, and the lumen was filled with follicular fluid (shown by black arrows) (Figures 8(a) and 8(c)). There was no significant change in the ovaries in the CATGNOG blank group (Figures 8(b) and 8(d)). In the blank control group of female rabbits, the uterine smooth muscle layer was thin, the inner ring was longitudinal, the gland structure was clear, and it is primarily round or oval (black arrow) (Figures 8(e) and 8(g)). There was no apparent change in the uterus of the CATGNOG blank group (Figures 8(f) and 8(h)). In the blank control group of male rabbits, the endogenous seminiferous tubules (black arrow) were densely arranged, the layers were transparent, and many sperm were found in the seminiferous tubules (Figures 8(i) and 8(k)). There was no significant difference between the gel and the control groups (Figures 8(j) and 8(l)). The epididymis of the male rabbit blank control group was composed of nonciliated cells and columnar ciliated cells. There were numerous cilia next to the cavity surface cells. The cavity surface cells were neat, the efferent tubule (black arrow) structure was clear, and there were several evenly stained sperms in the cavity (Figures 8(m) and 8(o)). No significant difference was observed between the gel preparation group and the blank control group (Figures 8(n) and 8(p)).

### 3.8. Skin Sensitization Test

#### 3.8.1. The Effect of CATGNOG on Skin Sensitization in *Cavia porcellus*

Six hours after administration, the skin sensitization of *Cavia porcellus* was scored. The results showed that CATGNOG had no sensitization effect. The sensitization rate of the positive control group was 100%. The results are shown in Table 27.

#### 3.8.2. The Effect of CATGNOG on Blood Routine in *Cavia porcellus*

There was no abnormal image in blood routine examination, which was not statistically significant compared with the blank group (*P* > 0.05). The results are shown in Table 28.

#### 3.8.3. The Effect of CATGNOG on the Viscera Index of *Cavia porcellus*

The viscera results of *Cavia porcellus* are shown in Table 29, which were not statistically significant compared with the control group (*P* > 0.05).

#### 3.8.4. HE of *Cavia porcellus* Reproductive Organs

**Figure 9:**
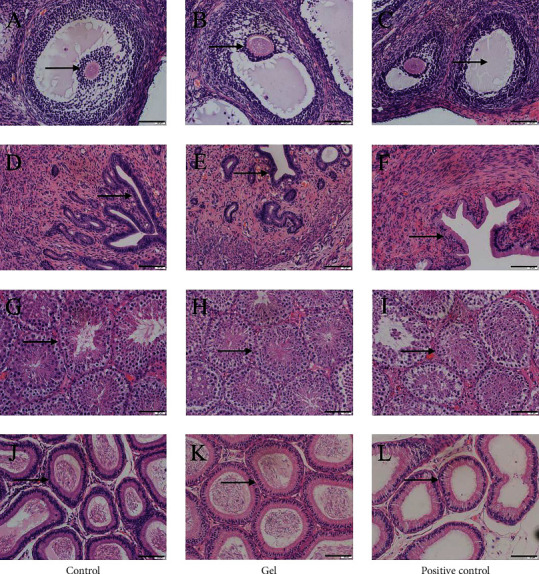
Effects of CATGNOG on the reproductive organs of *Cavia porcellus* (H&E, ×200). (A-C) Ovary. (D-H) Uterus. (G-I) Testis. (J-L) Epididymis. Scale bar: 50 *μ*m.

HE of reproductive organs of *Cavia porcellus* showed that there were many granulosa layers in the ovary of female *Cavia porcellus*, all follicles were visible, and the fluid in the lumen was abundant (black arrow) (Figure 9(a)). There was no obvious change in the ovary between the gel and the blank groups (Figure 9(b)). In the positive control group, the number of follicles decreased, the number of atresia follicles increased, and the ovaries tended to shrink (Figure 9(c)). In the blank control group, the epithelial cells of uterine mucosa were single-layer columnar cells, with the abundance of glands and clear structure (Figure 9(d)). Compared with the blank group, there was no significant difference in the uterus in the gel dose group (Figure 9(e)). There was pseudostratification in the upper mucosa in the positive control group, with significantly reduced glands (Figure 9(f)). In the blank control group of male *Cavia porcellus*, the testicular endogenous seminiferous tubules were densely arranged, all spermatogenic cells were visible, the layers were clear, and a large number of sperms were visible (Figure 9(g)). There was no significant difference between the gel dose group and the control group (Figure 9(h)). Some spermatogenic epithelial cells in the positive control group were disorganized, and the cells in the seminiferous tubules were irregularly arranged and loose (Figure 9(i)). In the blank control group, there were a large number of sperm cells with uniform staining in the lumen of the efferent tubules of the epididymis, the arrangement of principal cells was neat and orderly, and the cytoplasm was relatively full (Figures 9(j) and 9(k)). In the positive control group, the intercellular space of the efferent tubules was relatively large, and the intraluminal sperms were reduced (Figure 9(l)).

### 3.9. Study on Pharmacodynamics of CATGNOG Promoting Wound Healing

#### 3.9.1. Bacterial Count and Identification

**Figure 10:**
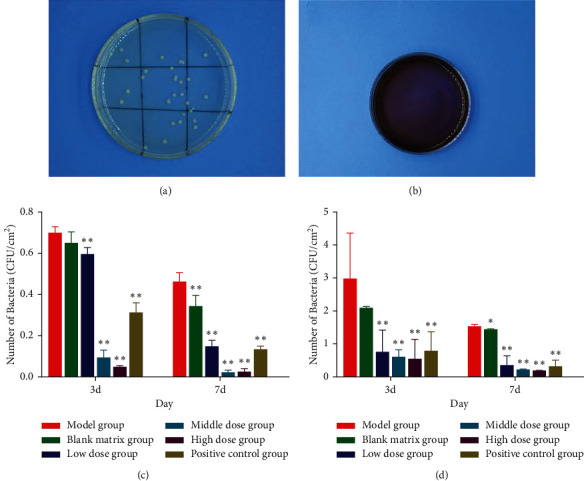
Bacterial count and identification (CFU/cm2, $\bar{X}\pm s$, *n* = 7). (a) The nutrient agar. (b) Eosin methylene blue. (c) The number of bacteria in normal rats. (d) The number of bacteria in diabetic rats. ^*∗*^*P* < 0.05; ^*∗∗*^*P* < 0.01 versus model group.

The colonies were beige on nutrient agar medium (Figure 10(a)) and no colonies on eosin methylene blue medium (Figure 10(b)). The results showed that the beige colony was gram-positive bacteria. After bacterial identification, gram staining showed that the colony was purple, which further confirmed that the colony was gram-positive bacteria.

The number of bacteria in the DM group was higher than that in the normal group, and the number of bacteria in both groups was less than 3 days in 7 days. As shown in Figure 10(c), the number of bacteria in the low-, medium-, and high-dose groups and the positive control group was lower than that in the model group (*P* < 0.01), and the number of bacteria in the blank control group was lower than that in the model group for 7 days (*P* < 0.01). Except for the blank control group, the number of bacteria in the other groups of diabetic rats was lower than that in the model group at 3 days (*P* < 0.01). The number of bacteria in the low-, medium-, and high-dose groups and the positive control group at 7 days was significantly lower than that in the model group (*P* < 0.01). There was a significant difference between the blank control group and the model group (*P* < 0.05), as shown in Figure 10(d).

#### 3.9.2. Effect of CATGNOG on Wound Healing Morphology in Normal Rats

**Figure 11:**
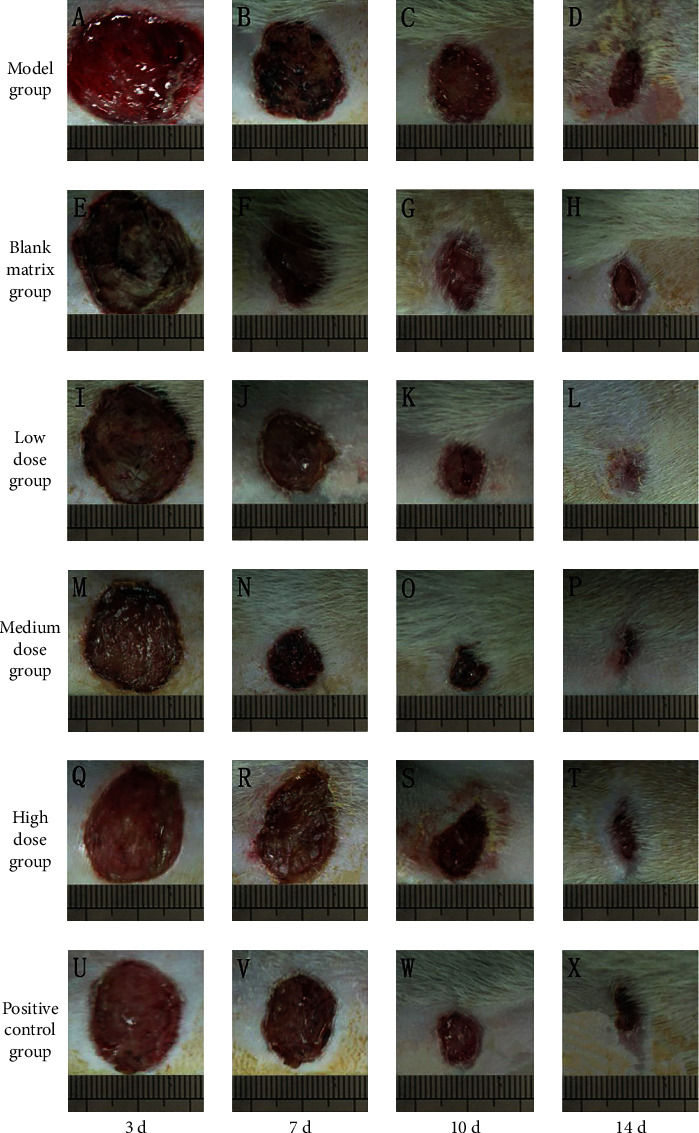
Change of wound surface of rats in the normal group at different administration time. (A–D) Model group. (E–H) Blank matrix group. (I–L) Low-dose group. (M–P) Medium-dose group. (Q–T) High-dose group. (U–X) Positive control group.

In the model group, the wound surface was bright red, the exudate was more, the granulation tissue formed less, and the wound healing rate was slower than that of other groups (Figures 11(a)–11(d)). On the 7th day, the wound in the blank matrix group was obviously reduced, and the granulation tissue growth was better than that in the model group (Figures 11(e)–11(h)). In the low-dose group, the wound healed quickly, most of the wounds were epithelialized on the 14th day, and the wounds were nearly closed on the 14th day (Figures 11(i)–11(l)). The wound healing rate of the medium-dose group was the fastest and covered with scab, and it was completely epithelialized on the 14th day (Figures 11(m)–11(p)). The wound of the high-dose group was reddish, and the healing speed was slower than that of the medium-dose group (Figures 11(q)–11(t)). In the positive control group, there was pus exudation on the 3rd day, and the granulation tissue of the wound grew well (Figures 11(u)–11(x)).

As shown in Table 30, 7 days after administration, the healing rate of the medium-dose group was higher than that of the model group and the blank matrix group (*P* < 0.05), which was significantly different from that of the low-dose group (*P* < 0.01). The healing rate of the positive control group was higher than that of the model group and low-dose group (*P* < 0.05). After 10 days of administration, the healing rate of the medium-dose group was higher than that of the model group (*P* < 0.05), while that of the high-dose group was higher than that of the medium-dose group (*P* < 0.05). At 14 days after administration, the healing rate of the low-dose group was higher than that of the model group (*P* < 0.05). The healing rate of the medium-dose group was higher than that of the model group, which was significantly higher than that in the model group and positive control group (*P* < 0.01). The healing rate of the high-dose group was higher than that of the low-dose group (*P* < 0.01).

#### 3.9.3. Effect of CATGNOG on Wound Healing Morphology of DCU

**Figure 12:**
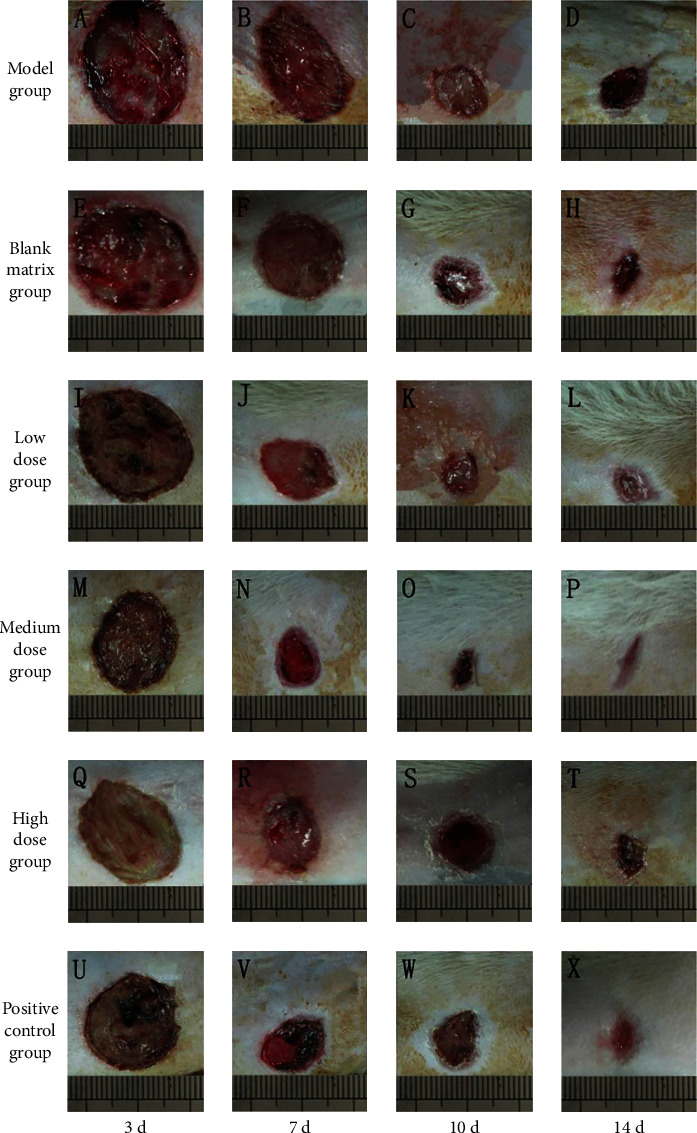
Change of wound surface of rats in the diabetic group at different administration time. (A–D) Model group. (E–H) Blank matrix group. (I–L) Low-dose group. (M–P) Medium-dose group. (Q–T) High-dose group. (U–X) Positive control group.

In the model group, the wound surface was bright red, and the wound healing rate was slower than that of other groups (Figures 12(a)–12(d)). In the blank matrix group, the wound surface was bright red at 3 days, white on the 7th day, and covered with black scab at 10 days (Figures 12(e)–12(h)). In the low-dose group, there was scab covering on the 3rd day, and thick fluid exuded at 7 days (Figures 12(i)–12(j)). In the medium-dose group, the healing rate was the fastest, the basal granulation was generated at 7 days, and the wound surface was completely epithelialized at 14 days (Figures 12(m)–12(p)). In the high-dose group, the wound surface healed slower, the wound surface was pale white, and there was pus exudation (Figures 12(q)–12(t)). The positive control group was covered with black scab, and the granulation was reddish at 14 days (Figures 12(u)–12(x)).

In diabetic rats, the healing rate in the medium-dose group was higher than that in other groups, as shown in Table 31. After 3 days of administration, the healing rate of the medium-dose group was higher than that of the model, blank matrix, and low-dose group (*P* < 0.05). The positive control group, blank matrix group, and low-dose groups had statistically significant differences with the model group (*P* < 0.05); 7 days after administration, there was a significant difference between the medium-dose group and blank matrix group (*P* < 0.05), there was a significant difference between medium-dose group and model group, blank matrix group, and low-dose group (*P* < 0.01), and the healing rate of the medium-dose group was higher than that of the model group and blank matrix group (*P* < 0.01). There was a significant difference between the positive control group and the blank matrix group (*P* < 0.01).

#### 3.9.4. Effect of CATGNOG on Pathological Structure of DCU Wounds

**Figure 13:**
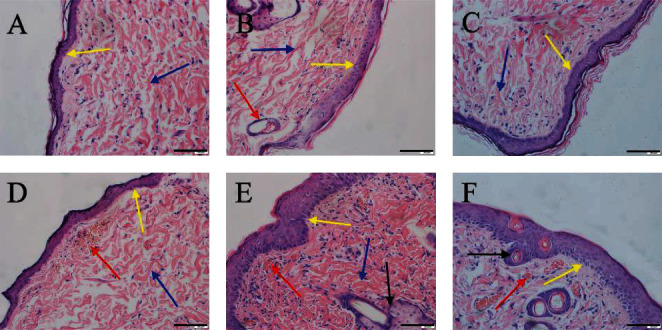
Histological comparison of wound granulation in DCU rats (H&E, ×200). (a) Model group. (b) Blank matrix group. (c) Low-dose group. (d) Medium-dose group. (e) High-dose group. (f) Positive control group. Scale bar: 50 *μ*m.

On the 14th day, a large number of collagen fibers (blue arrow) were seen in the diabetic model group, and the wound epithelium began to form (yellow arrow) (Figure 13(a)). In the blank matrix group, collagen was slender and disordered (blue arrow). Epithelialization (yellow arrow), superficial capillaries of dermis (red arrow), and sebaceous glands have been formed (Figure 13(b)). In the positive control group, stratum corneum increased, and epithelialization (yellow arrow) and collagen fibers disordered (blue arrow) (Figure 13(c)). In the low-dose group, massive dilation and hyperemia of capillaries (red arrow) and epidermis began to form (yellow arrow), and collagen fibers gathered (Figure 13(d)). In the medium-dose group, the hair follicles and sebaceous glands were hyperplastic (black arrow) and epithelialized, all layers of cells were clearly visible (yellow arrow), fibroblasts were dominant (blue arrow), the capillaries were dilated and congested (red arrow) (Figure 13(e)). In the high-dose group, there were telangiectasia, hyperemia (red arrow), hair follicle formation (black arrow), and epidermal epithelization (yellow arrow) (Figure 13(f)).

## 4. Discussion

Compared with other dosage forms, gel has the advantages of good spreadability, no greasy feeling, easy cleaning, comfort after coating, no tight feeling, good transdermal absorption, and good biocompatibility. Carbomer is often used as a matrix for gel, but the generation of NO in CATGNOG must be carried out under acidic conditions, while carbomer must be used in a neutral environment to form gels. Therefore, hydroxyethyl cellulose with good stability was chosen as a matrix for CATGNOG. Moisturizing agents can not only promote wound healing but also maintain certain stability in gel storage. In the choice of humectant, the moisturizing rate of glycerol is 32.30%, and its moisturizing effect is better than that of propylene glycol and polyethylene glycol. Therefore, glycerol is chosen as the moisturizer of gel. CATGNOG has high water content and is prone to mildew, requiring the addition of preservatives, and the commonly used preservative is 0.2% ethylparaben [40].

There are many evaluation indexes of gel. In order to prevent the one-sided results caused by a single evaluation index, sensory index, experimental index, and comprehensive experimental index of NO release time were selected to comprehensively evaluate the gel and choose the best formula. The comprehensive scores of prescription 1 and prescription 2 were 9.82 and 9.49, respectively, and the scores were close. In order to further study the screening of prescription and the permeability of gel, the *in vitro* transdermal absorption of intact skin and exfoliated skin was designed in this experiment. In the process of treating mouse skin, it is necessary to remove not only the fat and mucosal tissue but also subcutaneous blood vessels because the gel will penetrate into the blood vessels through the skin, which will affect the experimental results. The subcutaneous tissue of the abdomen is less than that of back skin, so the abdomen skin is selected as the carrier for transdermal absorption *in vitro*. After skin preparation, put in the refrigerator for later use. During the storage, it was found that the storage time affected the transdermal absorption of the drug, and the time should not be too long. Therefore, the skin should be prepared and stored every other day to minimize the operation error. The average temperature of the human body surface is 33.5°C. In order to avoid excessive deviation of experimental results, the temperature of the diffusion cell is 37.0°C, which is due to the low activity of mouse skin *in vitro*, and the increase of temperature is helpful to improve the activity. In the permeability study, the cumulative release per unit area of preparation 1 was superior to that of formulation 2 at various points, so we selected preparation 1 as the final formulation. Analysis of the release characteristics and kinetic model of preparation 1 showed that the release rate of CATGNOG acting on exfoliated skin was higher than that of intact skin.

CATGNOG is composed of gel 1 and gel 2. Gel 1 is the main drug of *Centella asiatica* total glycosides, so gel 1 is brownish-yellow semisolid, and gel 2 is a colorless and transparent semisolid gel with a certain fluidity. The results of the preliminary stability test of CATGNOG showed that the gel was relatively stable to high-speed centrifugation and low-speed centrifugation, and the appearance characteristics and content had no change. In the high-temperature test, the color, spread, viscosity, and total glycosides content of *Centella asiatica* changed at 5 days, so the gel should not be stored at a high temperature. In the accelerated test, the color, spreading property, and viscosity of gel 2 change at 30 days, so this gel should not be stored at 25°C. In the low-temperature test, the gel did not change for 90 days. Therefore, it is suggested that this gel should be stored at a low temperature of 4 ∼ 8°C.

CATGNOG is topical skin preparation. In order to study the safety of skin medication, it is necessary to carry out skin medication toxicity tests. At present, rats and rabbits are often taken as research objects in skin toxicity tests, and the safety of the gel can be judged by observing the toxic reaction of animals after local application. The reproductive system is susceptible to the influence of toxicants [41], so research on reproductive toxicity is indispensable. Viscera index is an objective, practical, and sensitive indicator in toxicology, which can directly reflect the comprehensive toxicity of viscera after medication and is an essential clue to the target organ of toxicant action; The decrease or increase of viscera index can reflect the atrophy or congestion of viscera [42]. The ovary is the central organ of the female reproductive system, and the uterus is the site of producing menstruation and giving birth to the fetus, which is the main target organ for the action of estrogen chemicals. Hence, the female reproductive system study is mainly the study of the ovary and uterus [43, 44]. The testes are vital organs that secrete male hormones and produce sperm, and the specific structures include the seminiferous tubules as well as the surrounding connective tissue [45]. In mammals, sperm need to pass through a special duct called the epididymis to mature and obtain fertilization ability after secretion from the testis [46].

The biological effect mechanism was expounded from the macroscopic individual, organ, tissue level and the microscopic cellular and molecular level, and the feasibility of the gel as a topical drug was discussed. The toxic reaction of the gel was observed in rats, rabbits, and guinea pigs by acute skin toxicity, skin irritation, and sensitization tests. During the experiment, the animals were in good health, with normal drinking water, food intake, excretion, and skin and hair color. HE staining was used to observe the effect of total glycosides of *Centella asiatica* on reproductive toxicity. Acute toxicity and skin irritation tests showed no obvious reproductive toxicity. In the allergy test, there was no obvious difference between the control group and the gel group, but there were slight changes in the reproductive tissues and organs between the positive control group and the control group. Whether 2, 4-dinitrochlorobenzene has an effect on the reproductive organs of guinea pigs needs to be confirmed by further studies.

DCU is one of the serious and intractable complications of diabetes. Intraperitoneal injection of STZ can directly destroy the islet *β* cells of rats, resulting in the disturbance of insulin signal transduction and glucose metabolism, and the characteristics of diabetes in rats. At present, the full-thickness skin defect model is the most convenient and accurate model to simulate the wound healing process of DCU. Therefore, this study established a diabetic rat model by intraperitoneal injection of STZ and made a full-thickness skin defect model to simulate the wound of DCU. The effects of CATGNOG on ulcer wound healing in normal and diabetic rats were investigated by monitoring and analyzing wound healing, wound granulation tissue structure, and corresponding parameters. The results showed that the number of bacteria in the high-, medium-, and low-dose groups of CATGNOG was lower than that in the model group (*P* < 0.05), which indicated that the preparation had an antibacterial effect. The number of bacterial colonies in the diabetic group was higher than that in the normal group, which was due to the low immunity of diabetic rats and the proliferation of bacteria. By observing the wound healing of skin ulcer, it was found that the wound healing rate of rats in the medium-dose group was higher than that in other groups, and the effect was the best. The wound healing rate in the high-dose group is lower than that in the medium-dose group. The possible reasons are as follows: (1) When the dose is high, the wound is tightly covered, and the poor permeability of the wound leads to slow wound healing. (2) In the process of administration, it was found that the higher the dose, the easier it was to form a dry scab. In order not to affect the secondary absorption of the drug, it is necessary to clean and wipe the wound every day. In this process, excessive cleaning may lead to wound tear in the high-dose group, which may affect wound healing. (3) The drug concentration in the high-dose group has exceeded the optimal concentration for ulcer skin repair. The specific reasons for the formation need to be further studied and revealed by the research group in the later stage.

## 5. Conclusions

In this study, *Centella asiatica* total glycosides and nitric oxide, which have a therapeutic effect on skin ulcers, were combined into a gel. The quality standards, skin toxicity, and reproductive toxicity of the gel were studied, and a new preparation for treating DCU was developed. One of the critical functions of the gel developed in this project is to quickly make NO adhere to the skin, increase its half-life and release it slowly after contact with the wound. Studies have shown that CATGNOG has no skin irritation, no skin sensitivity, no acute skin toxicity, and no reproductive toxicity. CATGNOG can inhibit the growth of bacteria and promote ulcer wound healing. In addition, the wound healing rate of 14 days in the medium-dose (8% *Centella asiatica* total glycosides) group of CATGNOG was the highest, which was the most effective in the treatment of DCU.

## Figures and Tables

**Table 1:** Gel sensory index and scoring standard.

Investigation index	Requirement	Grading standard	Score
Appearance characteristics	Transparent semisolid material.	Transparent and semisolid	2
		Slightly turbid color and poor transparency	1
		Very muddy color	0

Uniformity	The whole being uniform, no larger particles and clumps, and so on.	The whole being uniform and no larger particles and clumps	2
		A small number of larger particles and clumps	1
		A large number of large particles and clumps	0

Spreading property	Elastic, easy to smear, and smear the skin without any discomfort.	Elastic, easy to smear, and smear the skin without any discomfort	2
		Poor elasticity and slightly difficult to smear	1
		Difficult to evenly smear and smear the skin difficult to adapt	0

Centrifugal stability	3000 r/min centrifugal 30 min and no fragmentation.	No fragmentation	1
		Split phenomenon	0.5
		Serious stratification	0

Heat storage stability	The gel is placed in a 60°C drying oven for 24 h, and the gel is uniform.	No fragmentation	1
		Split phenomenon	0.5
		Serious stratification	0

Cold storage stability	The gel is placed in the refrigerator at −20°C for 24 h, and the gel is uniform and has no phenomenon.	No fragmentation	1
		Split phenomenon	0.5
		Serious stratification	0

Freeze-thaw stability	The gel is placed in the refrigerator with a −20°C of 24 h, and it is naturally thawed at room temperature.	No fragmentation	1
		Split phenomenon	0.5
		Serious stratification	0

*Note.* According to the actual observation results, the score of each item is 0–2. The total score is 10.

**Table 2:** Factors and levels of orthogonal test for forming process of CATGNOG.

Level	Factor (%)			
	A	B	C	D
	*Centella asiatica* total glycosides	Hydroxyethyl cellulose	Azone	Glycerol
1	8	2	1	5
2	12	2.4	2	7.5
3	16	2.8	3	10

**Table 3:** Standard of skin irritation response score.

Irritant condition		Score
Erythema		
	No erythema	0
	Barely visible	1
	Obviously visible	2
	Moderate or severe erythema	3
	Purple erythema and eschar	4
Edema		
	No edema	0
	Barely visible	1
	Visible	2
	Swelling of skin is about 1 mm; the outline is clear	3
	Swelling above 1 mm	4
The total score		8

**Table 4:** Standard of skin irritation evaluation criteria.

Average score	Evaluate
0～0.49	Nonirritating
0.5～2.99	Mild irritation
3.0～5.99	Moderate irritation
6.0～8.0	Strong irritation

**Table 5:** Standard of skin irritation evaluation criteria.

Sensitization rate (%)	Skin sensitization evaluation
0～10	No sensitization
11～30	Mild sensitization
31～60	Moderate sensitization
61～80	High sensitivity
81～100	Extreme sensitization

**Table 6:** Moisturizing effect of different moisturizers at 37°C.

Type	M (g)	M_1_ (g)	M_2_ (g)	Moisture retention (%)
Glycerol	24.8279	25.3279	25.1664	32.30
Propylene glycol	19.3942	19.8952	19.6728	44.39
Polyethylene glycol	23.5511	24.0263	23.8095	45.24

**Table 7:** Summary of hydroxyethyl cellulose forming study.

Way	Appearance	Spreading property	Stability			
			Centrifugal	Heat storage	Cold storage	Freeze-thaw
Swell	Good	Medium	Good	Medium	Good	Medium
Stir 0.5 h	Good	Bad	Good	Bad	Good	Medium
Stir 1 h	Good	Medium	Good	Bad	Good	Medium
Stir 1.5 h	Good	Good	Good	Bad	Good	Medium
Stir 2 h	Good	Good	Good	Good	Good	Good
Stir 2.5 h	Good	Good	Good	Bad	Good	Good

*Note.* The formability of hydroxyethyl cellulose is evaluated, and each evaluation index is divided into three grades: good, medium and bad.

**Table 8:** Orthogonal test and results of gel formation.

Test number		Factor				Molding score	Average	NO release time (min)	Composite score
		A	B	C	D				
1	Gel 1	1	1	1	1	9.40	9.05	40.00	9.82
	Gel 2					8.70			

2	Gel 1	1	2	2	2	8.40	8.30	40.67	9.49
	Gel 2					8.20			

3	Gel 1	1	3	3	3	7.40	7.45	41.00	9.07
	Gel 2					7.50			

4	Gel 1	2	1	2	3	8.40	8.25	34.00	8.65
	Gel 2					8.10			

5	Gel 1	2	2	3	1	8.30	8.25	36.00	8.90
	Gel 2					8.20			

6	Gel 1	2	3	1	2	9.30	9.15	34.70	9.19
	Gel 2					9.00			

7	Gel 1	3	1	3	2	8.20	7.95	34.70	8.57
	Gel 2					7.70			

8	Gel 1	3	2	1	3	8.50	8.65	29.00	8.26
	Gel 2					8.80			

9	Gel 1	3	3	2	1	8.60	8.65	32.70	8.50
	Gel 2					8.70			

**Table 9:** Precision results (*μ*g/mL).

	1	2	3	4	5	6	Average	RSD (%)
Asiaticoside	444.31	442.36	438.32	445.34	446.87	443.16	443.39	0.67
Madecassoside	224.57	230.4	232.43	222.38	224.91	229.09	227.30	1.57

**Table 10:** Repetitive results (*μ*g/mL).

	1	2	3	4	5	6	Average	RSD (%)
Asiaticoside	92.11	92.78	93.8	94.47	91.31	94.94	93.24	1.51
Madecassoside	297.49	302.94	322.76	303.16	303.71	312.04	307.01	2.94

**Table 11:** Stability study (*μ*g/mL).

	2 h	4 h	6 h	8 h	12 h	24 h	Average	RSD (%)
Asiaticoside	81.72	85.9	90.59	87.29	88.6	89.03	87.19	3.57
Madecassoside	255.4	275.06	266.02	264.01	265.1	271.2	266.13	2.53

**Table 12:** Recovery rate of asiaticoside.

Theoretical quantity (*μ*g)	Actual quantity (*μ*g)	Rate of recovery (%)	Average recovery (%)	RSD (%)
18.85	18.34	93.72	97.33	0.75
	19.10	103.13		
	18.27	92.77		
20.85	20.78	99.33		
	20.64	97.90		
	20.52	96.71		
22.85	22.64	98.31		
	21.96	92.58		
	23.03	101.56		

**Table 13:** Recovery rate of madecassoside.

Theoretical quantity (*μ*g)	Actual quantity (*μ*g)	Rate of recovery (%)	Average recovery (%)	RSD (%)
62.06	62.64	102.11	98.44	2.00
	62.78	100.82		
	62.15	98.49		
68.92	68.93	98.58		
	68.29	96.70		
	68.22	96.51		
75.78	74.12	94.76		
	74.15	94.82		
62.06	76.73	101.11		

**Table 14:** Cumulative release per unit area of *Centella asiatica* total glycosides in different prescription (*μ*g/cm^2^).

Time (h)	Intact skin		Exfoliated skin	
	Prescription 1	Prescription 2	Prescription 1	Prescription 2
3	86.8	19.77	548.5787	315.8171515
6	157.96	44.8	1313.66716	782.2929256
9	217.36	67.47	1869.66716	1256.954886
12	316.97	87.98	2296.699586	1696.691041
15	413.26	104	2623.949463	1986.39382
24	697.34	147.86	3717.414055	2948.724595

**Table 15:** The results of the equation of transdermal permeation of CATGNOG.

Skin	Fitting kinetic equation	*J* (*μ*g/cm^2^/h)	*R* ^2^	EF
Intact skin	*Q* = 0.0053t-0.0043	0.0053	0.9939	
	*Q* = 0.0348 t^1/2^–0.0056	0.0348	0.9474	

Exfoliated skin	*Q* = 0.0262 t+0.0711	0.0262	0.9726	4.943396226
	*Q* = 0.1779 t^1/2^–0.2039	0.1779	0.9987	5.112068966

**Table 16:** Results of gel pH.

Number	pH	Average	RSD (%)
1	4.65	4.67	0.61
2	4.65		
3	4.7		

**Table 17:** Determination of *Centella asiatica* total glycosides in gel.

Number	Asiaticoside (mg/g)	Madecassoside (mg/g)	*Centella asiatica* total glycosides (mg/g)	Average (mg/g)	RSD (%)
1	4.41	14.81	19.26	19.49	1.18
2	4.33	14.72	19.50		
3	2.56	15.24	19.72		

**Table 18:** Experimental results at high-temperature test.

Experimental condition	Placing time (d)	Appearance	Spreading property	Viscosity (mPa.s×1000)	Content (mg/g)	RSD (%)
*T* = 60°C	0	Gel 1, tan	Good	Gel 1, 10.5	19.12	9.25
		Gel 2, colorless		Gel 2, 15.25		
	5	Gel 1, color burn	Bad	Gel 1, 2	17.52	
		Gel 2, colorless		Gel 2, 0		
	10	Gel 1, color burn	Bad	Gel 1, 0.92	15.88	
		Gel 2, colorless		Gel 2, 0		

**Table 19:** Experimental results of accelerated test.

Experimental condition	Placing time (d)	Appearance	Spreading property	Viscosity (mPa.s×1000)	Content (mg/g)	RSD (%)
*T* = 25°C RH = 60%	0	Gel 1, tan	Good	Gel 1, 10.5	19.16	1.82
		Gel 2, colorless		Gel 2, 15.25		
	30	Gel1, color burn	Medium	Gel 1, 6.67	18.42	
		Gel 2, colorless		Gel 2, 0		
	60	Gel1, color burn	Medium	Gel 1, 3.5	18.56	
		Gel 2, colorless		Gel 2, 0		
	90	Gel 1, color burn	Bad	Gel 1, 1.08	18.95	
		Gel 2, colourless		Gel 2, 0		

**Table 20:** Experimental results at long term.

Experimental condition	Placing time (d)	Appearance	Spreading property	Viscosity (mPa.s×1000)	Content (mg/g)	RSD (%)
*T* = 4°C	0	Gel 1, tan	Good	Gel 1, 10.50	19.15	2.25
		Gel 2, colorless		Gel 2, 15.25		
	5	Gel 1, tan	Good	Gel 1, 11.67	19.32	
		Gel 2, colorless		Gel 2, 16.50		
	10	Gel 1, tan	Good	Gel 1, 10.40	19.76	
		Gel 2, colorless		Gel 2, 13.92		
	15	Gel 1, tan	Good	Gel 1, 11.83	18.59	
		Gel 2, colorless		Gel 2, 16.25		
	90	Gel 1, tan	Good	Gel 1, 10.25	18.97	
		Gel 2, colorless		Gel 2, 14.25		

**Table 21:** Effect of gel on the body weight of rats (g, $\bar{X}\pm s$, *n* = 4).

Group		n	Before	After administration of 7 d
Intact skin				
	Control	8	216 ± 19.90	235.625 ± 19.35
	High dose	8	211.8 ± 19.46	237.48 ± 24.89
	Medium dose	8	207.7 ± 25.02	225.25 ± 32.61
	Low dose	8	214.26 ± 24.48	238.29 ± 20.67

Breakage skin				
	Control	8	211.93 ± 16.21	228.84 ± 18.79
	High dose	8	220.93 ± 17.16	233.9 ± 23.23
	Medium dose	8	215.2 ± 12.15	241.24 ± 21.59
	Low dose	8	213.75 ± 20.20	225.75 ± 330.32

**Table 22:** Effect of gel on rat blood routine ($\bar{X}\pm s$, *n* = 4).

Group		WBC (×10^9^/L)	RBC (×10^12^/L)	HGB (g/L)	PLC (×10^9^/L)
Intact skin					
	Control	9.81 ± 2.35	7.80 ± 0.29	156.57 ± 6.50	1017.14 ± 256.00
	High dose	12.68 ± 4.35	8.11 ± 0.30	162.50 ± 5.07	992.00 ± 116.97
	Medium dose	13.92 ± 9.4	7.98 ± 0.0.52	157.00 ± 6.00	1112.60 ± 237.82
	Low dose	10.61 ± 4.01	7.63 ± 0.25	152.00 ± 6.44	989.20 ± 297.15

Breakage skin					
	Control	10.78 ± 3.47	7.82 ± 0.46	156.83 ± 10.42	1170.75 ± 117.74
	High dose	14.82 ± 4.81	7.89 ± 0.40	156.75 ± 11.71	1187.50 ± 149.19
	Medium dose	15.4 ± 5.42	8.00 ± 0.35	156.83 ± 46.08	1259.40 ± 181.12
	Low dose	10.47 ± 7.26	8.07 ± 0.58	159.71 ± 14.87	1143.00 ± 180.20

**Table 23:** Viscera index of rats (%, $\bar{X}\pm s$, *n* = 4).

Group		Ovary	Uterus	Epididymis	Testis
Intact skin					
	Control	0.08 ± 0.01	0.27 ± 0.07	1.17 ± 0.15	0.28 ± 0.05
	High dose	0.09 ± 0.02	0.28 ± 0.19	1.14 ± 0.10	0.28 ± 0.06
	Medium dose	0.09 ± 0.02	0.25 ± 0.03	1.32 ± 0.28	0.28 ± 0.03
	Low dose	0.07 ± 0.01	0.27 ± 0.06	1.20 ± 0.06	0.29 ± 0.06

Breakage skin					
	Control	0.08 ± 0.02	0.32 ± 0.10	1.30 ± 0.14	0.30 ± 0.03
	High dose	0.15 ± 0.09	0.23 ± 0.16	1.32 ± 0.13	0.32 ± 0.03
	Medium dose	0.08 ± 0.02	0.21 ± 0.03	1.21 ± 0.14	0.31 ± 0.05
	Low dose	0.07 ± 0.02	0.20 ± 0.03	1.39 ± 0.22	0.32 ± 0.07

**Table 24:** The average value of gel on rabbit skin irritation.

Group		n	Observation time			
			1 h	24 h	48 h	72 h
Intact skin						
	Control	4	0	0	0	0
	Gel	4	0.15	0.19	0.31	0

Breakage skin						
	Control	4	0	0	0	0
	Gel	4	0.20	0.23	0.32	0

**Table 25:** Effect of gel on rabbit blood routine ($\bar{X}\pm s$, *n* = 4).

Group		WBC (×10^9^/L)	RBC (×10^12^/L)	HGB (g/L)	PLC (×10^9^/L)
Intact skin					
	Control	8.96 ± 1.57	8.76 ± 0.64	139 ± 8.89	372 ± 46.79
	Gel	10.63 ± 0.57	8.59 ± 0.46	145 ± 5.61	415 ± 45.46

Breakage skin					
	Control	10.50 ± 1.12	6.84 ± 0.13	145.67 ± 4.93	396.33 ± 38.42
	Gel	8.86 ± 1.41	7.12 ± 0.58	149.50 ± 7.78	357.50 ± 35.36

**Table 26:** Viscera index of rabbit (%, $\bar{X}\pm s$, *n* = 4).

Organ	Intact skin group		Breakage skin group	
	Control	Gel	Control	Gel
Ovary	0.0086 ± 0.0032	0.0115 ± 0.0024	0.0141 ± 0.004	0.0127 ± 0.0027
Uterus	0.089 ± 0.0022	0.0816 ± 0.002	0.0859 ± 0.0158	0.0749 ± 0.016
Epididymis	0.0889 ± 0.0014	0.0934 ± 0.0011	0.088 ± 0.011	0.0975 ± 0.0117
Testis	0.0422 ± 0.0025	0.0398 ± 0.0022	0.0186 ± 0.0003	0.0197 ± 0.0013

**Table 27:** The mean value of allergic reaction of gel to *Cavia porcellus*.

Group	n	Reaction time				Sensitization rate (%)
		6 h	24 h	48 h	72 h	
Control	10	0	0	0	0	0
Positive	10	1.15	1.63	2.9	3.3	100
Gel	10	0	0	0	0	0

**Table 28:** Effect of gel on *Cavia porcellus* blood routine ($\bar{X}\pm s$, *n* = 10).

Group	WBC (×10^9^/L)	RBC (×10^12^/L)	HGB (g/L)	PLC (×10^9^/L)
Control	8.87 ± 3.79	5.87 ± 0.44	153.83 ± 3.97	552.17 ± 212.01
Positive	11.70 ± 7.89	6.16 ± 0.37	158 ± 14.23	572.17 ± 101.90
Gel	8.51 ± 7.34	6.11 ± 0.24	156.67 ± 5.03	612.6 ± 237.82

**Table 29:** Viscera index of *Cavia porcellus* (%, $\bar{X}\pm s$, *n* = 10).

Organ	Control group	Gel group	Positive group
Ovary	0.0252 ± 0.0025	0.0272 ± 0.0032	0.0261 ± 0.0047
Uterus	0.2209 ± 0.0037	0.22456 ± 0.0155	0.2187 ± 0.0222
Epididymis	0.4896 ± 0.1134	0.5420 ± 0.0668	0.4673 ± 0.0535
Testis	0.1510 ± 0.0341	0.1493 ± 0.0446	0.17094 ± 0.1063

**Table 30:** Wound healing rate of normal rats.

Group	Wound healing rate (%)			
	3 d	7 d	10 d	14 d
Model	37.08 ± 4.89	71.83 ± 11.01	88.55 ± 5.32	94.75 ± 3.12
Blank matrix	38.58 ± 5.78	72.31 ± 10.97	88.95 ± 7.28	96.19 ± 5.82
Low dose	46.15 ± 13.43	75.98 ± 3.58	90.53 ± 2.93	98.09 ± 0.54^Δ^
Medium dose	42.19 ± 7.67	83.45 ± 2.45^Δ^^*∗*##^	93.86 ± 2.91Δ	98.98 ± 0.91^ΔΔ#^
High dose	40.10 ± 6.46	78.39 ± 6.52	88.42 ± 4.04^☆^	96.151 ± 1.88^##^
Positive control	45.99 ± 12.26	82.55 ± 5.96^Δ#^	92.15 ± 3.53	97.02 ± 1.21^☆☆^

*Note.* ^Δ^ *P* < 0.05; ^ΔΔ^*P* < 0.01 versus the model group. ^*∗*^*P* < 0.05; ^*∗∗*^*P* < 0.01 versus the blank matrix group. ^#^*P* < 0.05; ^##^*P* < 0.01 versus the low-dose group. ^☆^*P* < 0.05; ^☆☆^*P* < 0.01 versus the medium-dose group.

**Table 31:** Wound healing rate of diabetic rats.

Group	Wound healing rate (%)			
	3 d	7 d	10 d	14 d
Model	27.12 ± 7.37	70.01 ± 4.81	77.92 ± 5.69	91.98 ± 2.85
Blank matrix	26.39 ± 9.24	68.66 ± 10.81	82.21 ± 6.61	92.30 ± 3.69
Low dose	25.20 ± 6.70	76.27 ± 9.80	86.21 ± 3.96^ΔΔ^	94.78 ± 4.86
Medium dose	46.86 ± 14.31^ΔΔ^^*∗∗*##^	79.78 ± 8.24^Δ^	93.18 ± 2.98^ΔΔ^^*∗∗*##^	98.70 ± 1.18^ΔΔ∗∗^
High dose	28.75 ± 9.11^☆^	71.39 ± 14.80	81.28 ± 15.51	92.39 ± 8.27
Positive control	39.74 ± 11.71^Δ^^*∗*#^	78.83 ± 14.56	93.80 ± 5.56^ΔΔ*∗∗*^	97.81 ± 3.54^ΔΔ^

*Note.* ^Δ^ *P* < 0.05; ^ΔΔ^*P* < 0.01 versus the model group. ^*∗*^*P* < 0.05; ^*∗∗*^*P* < 0.01 versus the blank matrix; ^#^*P* < 0.05; ^##^*P* < 0.01 versus the low-dose group. ^☆^*P* < 0.05; ^☆☆^*P* < 0.01 versus the medium-dose group.

## Data Availability

The data presented in this study are available on request from the corresponding author.
